# The Bacillus of Plague

**Published:** 1898-01-29

**Authors:** 


### THE BACILLUS OF PLAGUE.

A very complete and useful article on the bacillus of plague appears in *Science Progress* from the pen of Dr. Buckmaster. He points out that, during the forty years from 1855 to 1895, the plague has appeared more than sixty times in large or small epidemics, at least six or seven of these outbreaks being in India. Kitasato of Tokio discovered the specific microbe of plague on June 14th, 1894, and a few weeks later Yersin made the same discovery independently. Some difference of opinion has arisen as to whether the bacillus of Kitasato is identical with that of Yersin, but the majority of bacteriologists regard them as the same. The pest-bacillus is easily cultivated outside the body, and, indeed, grows well on all varieties of media. It is easily killed by drying or by weak solutions of disinfectants like carbolic acid, lyssol, mercury perchloride, and milk of lime. Heat also kills; even a temperature of 50 deg. Centigrade causing the death of all pest-bacilli exposed to it for an hour. As is well known, plague affects many animals, and all recent authors confirm the old observation that, when it attacks rats and mice, it soon also becomes a disease of man. It is also to be noted that, while the bacillus can easily be attenuated under cultivation, it acquires increased virulence when passed through a series of animals. What is the exact connection between epidemics affecting animals and those affecting men does not appear to be quite understood, for Hankin has shown that in some districts the plague has run its course without rats being affected, while in the neighbourhood of Hurdwar a genuine outbreak occurred among rats and terminated without spreading to the community. Flies also die of plague, and an infected fly has been shown to be capable of establishing the disease in a guinea-pig. Curiously enough, ants do not die of plague, although they rapidly eat up rats that have died of the disease. The practical application of bacteriological knowledge has taken two directions in regard to our dealings with the disease. Haffkine vaccinates against the plague by means of a fluid preparation obtained from dead cultures of the bacilli; while Yersin injects the anti-toxic serum of horses that have been immunised by repeated injection of cultures of the pest-bacillus.

